# Genetics, structure, and prevalence of FP967 (CDC Triffid) T-DNA in flax

**DOI:** 10.1186/s40064-015-0923-9

**Published:** 2015-03-26

**Authors:** Lester Young, Joseph Hammerlindl, Vivijan Babic, Jamille McLeod, Andrew Sharpe, Chad Matsalla, Faouzi Bekkaoui, Leigh Marquess, Helen M Booker

**Affiliations:** Department of Plant Sciences, 51 Campus Drive, University of Saskatchewan, Saskatoon, Saskatchewan S7N 5A8 Canada; National Research Council – Saskatoon, 110 Gymnasium Place, Saskatoon, Saskatchewan S7N 0W9 Canada; Quantum BioSciences, 101 – 100 Research Drive, Saskatoon, Saskatchewan Canada

**Keywords:** FP967, CDC Triffid, T-DNA, Flaxseed, *Linum usitatissimum*

## Abstract

The detection of T-DNA from a genetically modified flaxseed line (FP967, formally CDC Triffid) in a shipment of Canadian flaxseed exported to Europe resulted in a large decrease in the amount of flax planted in Canada. The Canadian flaxseed industry undertook major changes to ensure the removal of FP967 from the supply chain. This study aimed to resolve the genetics and structure of the FP967 transfer DNA (T-DNA).

The FP967 T-DNA is thought to be inserted in at single genomic locus. The junction between the T-DNA and genomic DNA consisted of two inverted Right Borders with no Left Border (LB) flanking genomic DNA sequences recovered. This information was used to develop an event-specific quantitative PCR (qPCR) assay. This assay and an existing assay specific to the T-DNA construct were used to determine the genetics and prevalence of the FP967 T-DNA. These data supported the hypothesis that the T-DNA is present at a single location in the genome.

The FP967 T-DNA is present at a low level (between 0.01 and 0.1%) in breeder seed lots from 2009 and 2010. None of the 11,000 and 16,000 lines selected for advancement through the Flax Breeding Program in 2010 and 2011, respectively, tested positive for the FP967 T-DNA, however.

Most of the FP967 T-DNA sequence was resolved via PCR cloning and next generation sequencing. A 3,720 bp duplication of an internal portion of the T-DNA (including a Right Border) was discovered between the flanking genomic DNA and the LB. An event-specific assay, SAT2-LB, was developed for the junction between this repeat and the LB.

## Introduction

In April 2009, transgenic flaxseed was detected in two 5,000 tonne shipments of flax during preprocessing in Europe (Flax Council of Canada 2009a). Shipments of Canadian flax have also tested positive for transgenes in Japan and Brazil (Flax Council of Canada 2009b). The EU and these countries have regulations regarding the detection of genetically modified (GM) flax in shipments and have zero tolerance for its presence. As a consequence, Canadian flax shipments to Europe fell dramatically from 2010–2012; Europe imported 80% of Canadian flaxseed prior to 2009 but only 20% in 2011 (personal communication, William Hill, President, Flax Council of Canada).

The Canadian industry responded to this reduction in flax exports to Europe by introducing measures to eliminate the presence of GM flax. These included the reconstitution of popular flax varieties (CDC Bethune, CDC Sorrel) from reserved GM-free seed stocks; encouragement by commodity groups to sell stored grain, clean out grain bins, and purchase fresh seed; and increased screening for the GM construct at all levels of production (Booker and Lamb 2012). As a consequence, the estimated incidence of the GM construct has fallen from a high of 0.004% in the 2009 and 2010 crop years to 0.0001% in the 2012 and 2013 crop years (Booker et al. 2014).

Positive results for GM flax presence in some Crop Development Centre (CDC; University of Saskatchewan, Saskatoon, Canada) breeder seed lots occurred when testing was done at or below the 0.01% level (CDC 2010). The Flax Council of Canada has proposed testing all certified seed for the presence of GM before it is used for planting (Flax Council of Canada 2011). Further development of valid methods to detect transgenes in flax and vigorous screening of flax breeding material for transgenes is urgently needed and will contribute towards understanding the inheritance of transgenes in the flax germplasm and, importantly, the restoration of export markets.

The presence of transgenes in flax shipments from Canada relates to the mid-1990s introduction of GM line FP967 (registered as CDC Triffid in 1997) (McHughen and Holm, 1994; McHughen et al. 1997). This variety was deregistered in 2001 due to concerns about the effect of production of GM flax on export markets (Ryan and Smyth 2012).

Here, we describe the development of an event-specific assay to detect the presence of FP967 transfer DNA (T-DNA). The assay developed was used to determine the number of loci containing the T-DNA in the FP967 genome. In addition, a retrospective look at breeder seed produced by the CDC to isolate potential sources of the GM construct was performed, as well as a detailed examination of lines currently passing through the CDC’s Flax Breeding Program. Cloning of genomic fragments from the T-DNA and next generation sequencing (NGS) of the FP967 line were done to obtain better knowledge of the GM construct.

## Materials and methods

### Sampling of breeder seed

A total of 27 samples of CDC flax breeder seed were obtained for registered CDC flax varieties. Subsamples were taken from each of these samples. Each subsample weighed approximately 5.7 g and contained about 950 seeds. A positive control sample, consisting of approximately 5.7 g of CDC Bethune seed spiked with a single heat-killed FP967 seed, was incorporated in each set of extracts. Between three and nine subsamples were examined from each sample of seed, with the number varying due to availability.

### DNA extraction breeder seed

DNA was extracted from these subsamples using a modified guanidinium hydrochloride-silica matrix extraction protocol. Whole seeds were ground at 1200 rpm for 4 min using a vertical ball mill (Genogrinder from Spex SamplePrep, Metuchen, NJ, USA) in 20 mL of 5 M NaCl with three 12 mm diameter zirconium-yttrium ceramic cylinders (Inframat Advanced Materials, Manchester, CT, USA) in a 50 mL tube. The resulting homogenate was centrifuged at 1250 × G for 10 min and 2 × 1.2 mL of supernatant drawn off and transferred to fresh tubes containing 20 μL of SilMag slurry (silica-coated iron powder, Chemicell GmbH, Berlin, Germany). The mixture was left to stand at room temperature, with occasional agitation, for 5 min. Subsequent washing and elution steps were performed using a magnetic rack (for tubes). The iron particles were collected and washed once with 1.2 mL of 4 M guanidinium hydrochloride in 50% ethanol and twice with 10 mM Tris HCl, pH 8.0 in 80% ethanol. Excess wash solution was drawn off using a pipette and the pellet allowed to air dry for 10–15 min. DNA was eluted in 100 uL sterile distilled water.

### Construct- and event-specific assays

**Figure 1:**
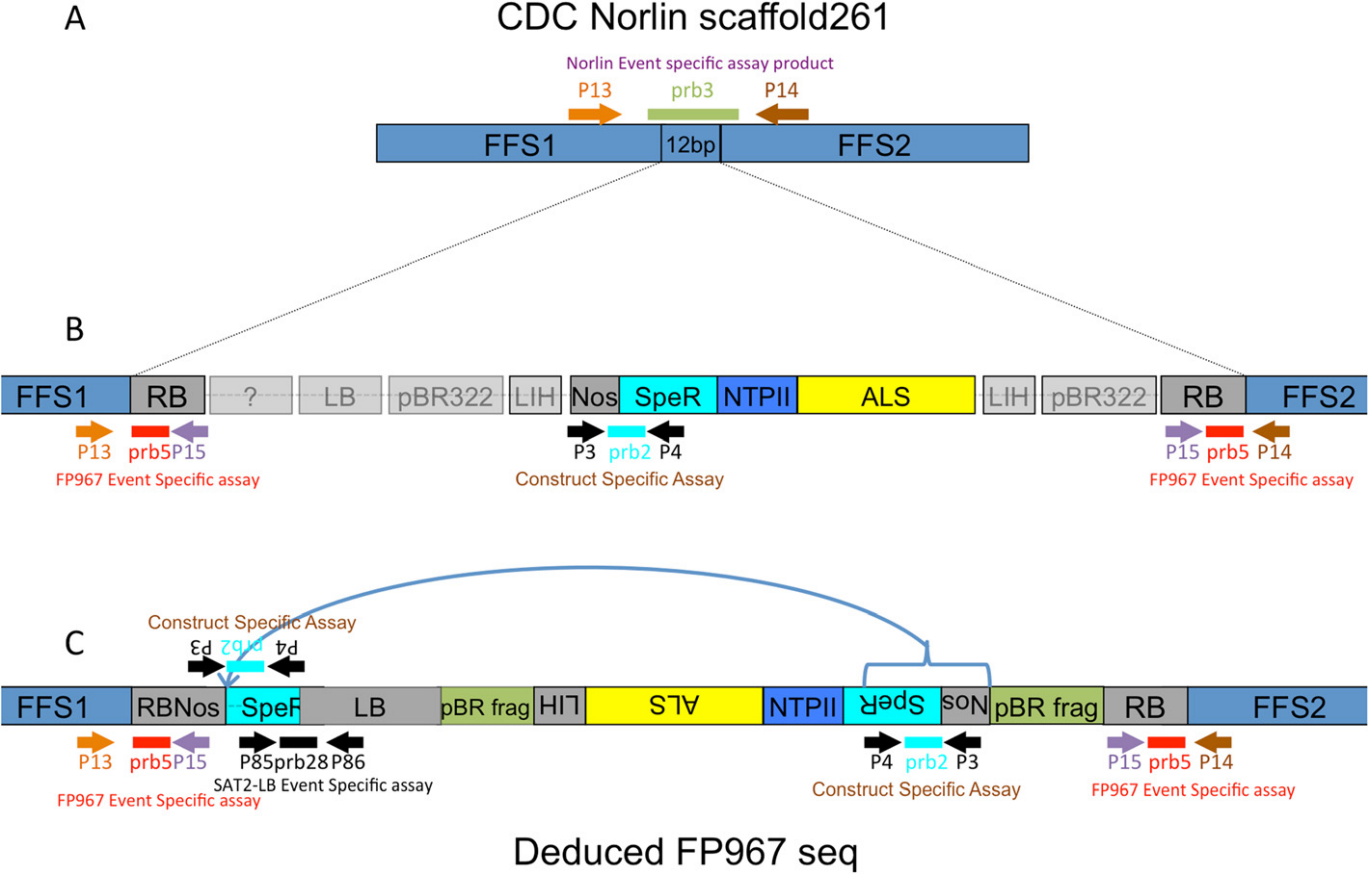
**Cartoon of the insertion of the FP967 T-DNA into scaffold 261 of the flax genome. A)** The insertion site of the FP967 TDNA into Norlin gDNA at scaffold261. The event specific assay detects uninterrupted gDNA from Norlin using primers P13 and P14 and Taqman prb3. See Tables 1 and 2 for more details. **B)** Known (in colour) and unknown (opaque grey) portions of the FP967 TDNA at the beginning of the project. The sequences and orientations of the LB and flanking region, the pBR322 fragments and the LIH had not been confirmed. The construct specific assay, which detects the DHFR fragment form E. coli and the Nos terminator, is indicated (P3, P4 and prb2). The event specific assay, developed in this project, is also shown. It uses a primer in Norlin gDNA (P13 or P14), a primer in the RB (P15) and a probe in the RB (prb5) to detect the TDNA. **C)** Deduced T-DNA structure after NGS and PCR fragment cloning. The inverted portion of the TDNA inserted between FFS1 and the LB is indicated, as is the new event-specific assay, which spans the junction between the SAT2 gene of the SpecR cassette and the LB (P85, P86 and prb28). The orientation and sequence of the LIH, AtALS, NPTII, SpecR cassette and internal Nos gene were deduced. Inverted sections were found to be oriented in the reverse direction.

Two Taqman assays were used to determine the presence of the FP967 T-DNA (Tables 1 and 2, Figure 1). The first assay is construct specific and is used in commercial testing laboratories to detect the presence of FP967 (Anon 2009; Grohmann et al. 2011). It consists of a pair of primers (Table 2) designed to amplify a 105 bp *NOS*-terminator to DHFR fragment of the FP967 T-DNA. A second pair of primers and a probe are used to detect a 68 bp fragment of stearoyl-acyl carrier protein desaturase (*SAD*) as a reference gene.

**Table 1:** **Use of the construct and event specific event assays to detect the FP967 TDNA**

	**Norlin**	**FP967 TDNA**	**Hemizygote**
Construct specific assay			
P3 + P4 + probe2		X	X
P1 + P2 + probe1 (detects SAD reference gene)	X	X	X
Event specific assay			
P13 + P14 + probe3	X		X
P13 + P15 + probe 5 and P14 + P15 + probe 5		X	X

**Figure 2:**
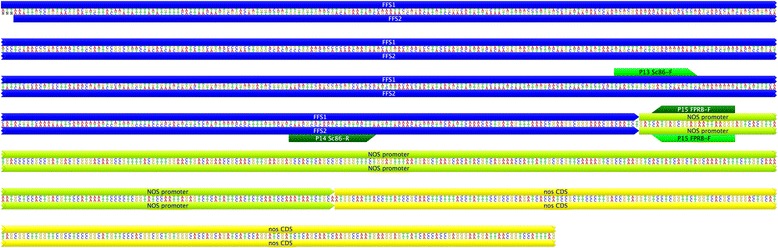
**Sequence of the two flanking regions of genomic DNA (FFS1 and FFS2) joining the T-DNA right border region.** The flanking sequences (FFS1 and FFS2, blue) are separated by 12 bp in a single fragment of flax genomic DNA; however, in this diagram FFS2 has been reverse complemented to demonstrate that the sequences of the two *NOS* gene fragments (green and yellow) are identical. Primers for the event specific assay (P13, P14 and P15) are indicated.

The construct specific assay is able to distinguish between WT and transgenic plants, while the event specific assay can also distinguish between hemizygous and homozygous individuals. Sequences of the primers and probes are shown in Table 2, while their approximate location is shown on Figure 2.

**Table 2:** **Primer and probe sequences used in the construct and event specific promoters**

**Primer/probe name**	**Sequence**	**Tm**	**Notes**
SAD reference: Amplifies 68 bp of stearoyl-acyl carrier protein desaturase			
P1	GCTCAACCCAGTCACCACCT	63	
P2	TGCGAGGAGATCTGGAGGAG	61	
prb1	TGTTGAGGGAGCGTGTTGAAGGGA	68	
Construct specific assay: Used by commercial testing companies to detect 105 bp of FP967 TDNA			
P3	AGCGCGCAAACTAGGATAAA	52	In NOS 3’terminator
P4	ACCTTCCGGCTCGATGTCTA	55	In DHFR gene of E.coli SpecR cassette
prb2	CGCGCGCGGTGTCATCTATG	67	
Event Specific assay: Detects WT gDNA at scaffold261 or the FP967 TDNA RB-flanking scaffold86 junction			
P13	CTATCGTCTGACTCTGACTG	49	FFS1 flax genomic region
P14	CAACGCCCACTCTCTTTCTTA	52	FFS2 flax genomic region
P15	CCCTTAATTCTCCGCTCATGATCAG	50	RB region of TDNA
prb3	CACTTCTTCAATTTTATTTCAATATGTCTTTC	60	Specific for FP967 TDNA insertion site on scaffold86
prb5	ATCAAACACTGATAGTTTAAACTGAAGGCGGG	67	Specific for RB region of TDNA
Event specific assay SAT2-LB: Detects internal inverted repeat of E. coli SpecR cassette adjacent to LB			
P85	TACATTAAAAACGTCCGCAATGTG	59	Located in LB
P86	CCTGCTCAGGGATCACCGAA	62	In SAT of E.Coli SpecR cassette
prb28	TATCCTGCCAAAAGCCGCGCCA	68	LB/Spec junction

The second assay was designed as an event-specific assay (Tables 1 and 2). Primers (P13, P14, and P15) were designed using inverse-PCR generated sequence of the right border region of the T-DNA (Figures 1 and 2). The presence of the T-DNA produces two fragments of 215 and 186 bp from FFS1 and FFS2, respectively. Both transgenic fragments are detected with the same probe. If the T-DNA is absent, a 202 bp fragment of scaffold261 is amplified and detected with a probe complementary to the region of DNA eliminated by insertion of the T-DNA. In this assay, homozygous plants are detected by one or the other probe while hemizygous plants produce both fragments (Table 1). Assay conditions are provided in the supplementary data archive (http://dx.doi.org/10.6070/H498851J).

### Sampling of breeding lines

Two seeds from each line selected for advancement through the CDC Flax Breeding Program in 2010 were grown on cotton in 96-well trays in the lab for 7–9 d. Each hypocotyl section was excised and hydrolyzed in 40 μL of 0.25 M NaOH at 95°C for 45 s before being neutralized with 60 μL of 0.5 M Tris pH 8.0. A second incubation at 95°C for 3 min was performed and the extracts allowed to cool to room temperature. A one microliter aliquot of each extract was used in the construct-specific assay (16 μL total volume). A single FP967 seedling was used as a positive control in each tray.

A different protocol was used on seed from the 2011 nursery. Ten seeds from each line selected for advancement through the program were homogenized in 4 M guanidine hydrochloride in 50% ethanol by a 5 mm zirconium-yttrium ceramic bead in a deep well microtitre plate using a vertical ball mill. Debris was pelleted by centrifugation at 3700 rpm for 10 min and 700 μL drawn off to a fresh deep well microtitre plate containing 15 μL of MagSil slurry. The subsequent wash steps were performed using a Kingfisher apparatus (ThermoFisher) and followed the same procedures as described for the bulk seed DNA extraction. A single microliter of the DNA extract was used in both the construct-specific and event-specific assays. A single heat-killed FP967 seed, along with nine CDC Bethune seeds, was used as a positive control in each plate.

The original papers describing the development of FP967 did not definitively state the number of copies of the T-DNA present in the line (McHughen 1989; McSheffrey et al. 1992). Accordingly, we used both qPCR assays to determine the number of copies of the T-DNA present within FP967. Both F2 and BC2 lines from Norlin × FP967 crosses were examined.

### Determining the sequence of the FP967 T-DNA

We used both PCR-cloning and NGS approaches to determine the sequence of the FP967 T-DNA. PCR primers were designed using a putative T-DNA structure, based on the design of the original construct (Zambryski et al. 1983; Fraley et al. 1985; Sanders et al. 1987; McHughen 1989; Sathasivan et al. 1990). Primers were designed using pTiC58 plasmid (GeneBank accession AE007871.2), T-DNA and *NOS* gene, *Arabidopsis acetolactate synthase* (NP_001189794.1), pBR322 (J01749.1), *neomycin phosphotransferase II* (AY909580.1), and the *E. coli spectinomycin resistance/dihydrofolate reductase* (SpecR/ DHFR) region used in the construction of pMON200 (Figure 1). Long PCR fragments generated from FP967 genomic DNA were generated using Phusion polymerase (NEB) and cloned into pBluescript. DNA Services, National Research Council – Saskatoon, performed Sanger sequencing of fragments. Alignment of clone sequences was performed using Geneious v 6.0 (Biomatters, http://www.geneious.com).

Genomic DNA was extracted from FP967 root tips and sequenced using Illumina HiSeq chemistry. Both mate-paired and paired-end reactions were performed, using 36 and 100 cycles, respectively. The resultant reads were assembled against the flax reference genome (version 1.0 at Phytozome.net, accessed 8 Feb 2015; Wang et al. 2012) and unaligned reads identified. Bowtie2 and Ray were used to assemble scaffolds and construct putative sequences for the FP967 T-DNA. In some instances, a reduced dataset consisting of reads not aligning with the *L. usitatissimum* reference sequence, collected using Bowtie2 (Boisvert et al. 2010), was used in *de novo* assembly using Ray (Langmead and Salzberg 2012) or SOAPdenovo. A wide range of kmers and parameters were used to remove the majority of non-relevant reads from the data. In addition to this approach, reads were aligned against known fragments of the T-DNA and the PCR-generated clone sequences using Bowtie2 (Fraley et al. 1983). Assembly and alignment of the reads and contigs were performed on a local machine using Geneious or using Hermes, a WestGrid server that is a part of Compute Canada Calcul Canada.

## Results and discussion

### Development of an event-specific assay for FP967 T-DNA

Inverse PCR from the Right Border (RB) of the T-DNA showed two copies of this sequence present in scaffold261 from the *Linum usitatissimum* genome assembly v1.0 (Figure 2). These two RB fragments were in an inverted orientation relative to one another and replaced 12 bp of scaffold261. Attempts to identify genomic regions adjacent to the Left Border (LB) were unsuccessful. These results suggest at least one insertion of the T-DNA or a duplication of a fragment of the T-DNA occurred. The propensity of T-DNA to undergo rearrangement and/or duplication, including inverted repeats, has been noted previously, e.g., (De Buck et al. 1999).

The inverted and repeated structure of the T-DNA RBs means that an event-specific assay could be developed. qPCR primers and probes were developed (Table 2, Figures 1 and 2) to detect both the intact Norlin scaffold261 sequence (named FFS1 and FFS2 for FP967 Flanking Sequence 1 and 2 on either side of the insertion site, respectively) as well as simultaneously detect both RB sequences. The event-specific assay is designed to produce one of two mutually exclusive products in homozygous individuals; that is, either the scaffold261 or the FFS/RB PCR product is synthesized and detected. In heterozygous individuals, or DNA admixtures containing both scaffold261 and the T-DNA, both products are amplified and detected. The detection of these two DNA fragments occurs in a single tube. The event-specific qPCR assay is complementary to the construct-specific assay used in commercial test laboratories (Anon 2009; Grohmann et al. 2011) and the assay designed to detect the mutated *Arabidopsis acetolactate synthase* (AtALS) gene that provides resistance to sulphonylurea herbicides (Nakamura et al. 2010).

The event-specific assay was tested on genomic DNA extracted from individual FP967 plants as well as from mixtures of FP967 and CDC Bethune seeds. The developed assay was specific to FP967 and could detect the T-DNA in DNA extracted from seed admixtures (results not shown). In our hands, the event-specific assay was sensitive to one FP967 seed in admixtures containing ~5,000 seeds. The described event-specific assay is not as sensitive as the construct-specific assay. The construct specific promoter may be more sensitive than the event specific assay as it was discovered that there is a duplication of the Nos gene-Streptomycin resistance cassette region of the TDNA containing the target of the construct specific assay (amplified by P3, P4 and prb2). This means that double the number of copies of the construct specific assay target are present.

The event-specific assay has the advantage of having one less primer than the commercial test. The assay also produces both a positive and a negative polymerase chain reaction (PCR) product, eliminating the need for a PCR control reaction (stearoyl-acyl carrier protein desaturase (SAD) in the commercial tests). As a result, this assay halves the number of PCR assays required compared to assays where the T-DNA and *SAD* are detected in separate reactions. However, because the event-specific assay is not as sensitive as the commercial assay, it is not as useful for detecting the presence of FP967 T-DNA in bulk samples.

### Genetics and prevalence of the FP967 T-DNA

The event-specific assay may be used to detect heterozygous individuals as it produces a PCR product from both the T-DNA and the non-transformed genomic DNA sequence. The inheritance of FP967 T-DNA was investigated using both the event- and construct-specific assays. Using the construct-specific assay, we detected the presence of FP967 T-DNA in 55 of 80 F_2_ plants, suggesting a single locus for the T-DNA (χ^2^ = 1.67, 1df, p =0.13). Using the event-specific assay to test the same F_2_ individuals, we observed 24 FP967 homozygotes, 31 heterozygotes, and 25 Norlin homozygotes in the same population, once again suggesting a single T-DNA locus (χ^2^ = 4.08, 2df, p = 0.07). This conclusion is further supported by the 15:17 ratio (T-DNA with scaffold261: scaffold261) of individuals in the BC1 generation (χ^2^ = 0.13, 1df, p =0.47). The results from these two independent populations indicate that a single insertion of the T-DNA is present in the Norlin genome, supporting previous work based on progeny tolerance to chlorosulfuron *in vitro* (McHughen 1989). Our results also clarify previous work using Southern blots that suggests one to three copies of the T-DNA are present in the FP967 genome (personal communication, Alan McHughen, University of California Center Sacramento). These results, along with the cloning and sequencing of the T-DNA strongly support the hypothesis that the T-DNA is present at a single locus in FP967.

The event- and construct-specific assays were used to detect the presence of the FP967 T-DNA in CDC breeder seed. A total of two assays tested positive for FP967 T-DNA in breeder seed sampled in 2009 (out of 45 performed over 15 samples) and three in breeder seed sampled in 2010 (out of 72 performed over 12 samples). The positive samples corresponded to CDC Arras and CDC Normandy. Possible trace levels of FP967 T-DNA were detected in a sample of CDC Mons breeder seed; however, this result was not replicated in repeated assays of the extracted DNA (Table 3). Breeder seed from other varieties (CDC Bethune, CDC Gold, CDC Sanctuary, CDC Sorrel, CDC Valour, Flanders, Somme, and Vimy) were negative for the T-DNA in our tests (Table 3); however, commercial testing of these sources returned positive results (Lamb and Booker 2011). The discrepancy between in-house and commercial testing results may be due to differences in sensitivity and the size of the replicates assayed. Commercial testing currently examines four 60 g replicates and records a positive result if the T-DNA is detected in a single replicate; the subsample size used in this study was much smaller. As the level of FP967 present in these seed batches is low, it is likely that the smaller sample size used in our in-house assays simply did not contain any FP967 seeds, thus making providing a negative result. Overall, the level of FP967 present in the breeder seed samples tested was low, between the 0.1% threshold detectable in the event-specific assay and the 0.01% standard of the commercial assay. As the level of FP967 is so low, modifications to our in house sampling procedure are required.

**Table 3:** **Summary of results for in-house and commercial testing of CDC flax breeder seed**

**Variety**	**Year sampled**	**# samples**	**# indep. in-house 5.7 g assays**	**Positive in-house assays**	**# indep. commercial 60 g subsamples**	**Positive commercial assays**
CDC Arras	2009	1	3	2	5	-
	2010	2	8	0	8	-
CDC Bethune	2009	3	9	0	15	-
CDC Gold	2009	1	3	0	1	-
CDC Mons	2009	1	3	0	2	+
	2010	5	16	trace	36	+
CDC Normandy	2009	1	3	0	2	+
	2010	5	14	3	36	+
CDC Sanctuary	2009	2	6	0	9	-
CDC Sorrel	2009	2	6	0	9	-
CDC Valour	2009	1	3	0	1	-
Flanders	2009	1	3	1	4	-
Somme	2009	1	3	0	4	-
Vimy	2009	1	3	0	1	-

A number of seed samples of CDC varieties were sampled in 2009 and 2010. These samples were tested at a small scale (5.7 g) in-house using the construct- and event-specific assays. Commercial laboratories using the construct-specific assay also tested these same samples. The commercial assay consisted of either a single or four independent 60 g subsamples. Results from the commercial tests were reported as either positive/negative for the subsamples as a whole, or positive/negative/trace for each subsample. As some four-subsample tests were reported as positive or negative, it is not possible to report the number of positive assays. The single positive in-house assay for CDC Mons was very weak and is reported as trace.

Breeding lines currently advancing through the CDC Flax Breeding Program were tested for the presence of the construct found in CDC Triffid to prevent further introduction of the FP967 T-DNA into the Canadian flaxseed supply. Lines selected for advancement through the CDC Flax Breeding Program have been examined since 2010. Approximately 11,000 and 16,000 lines were examined in 2010 and 2011, respectively, for the presence of the FP967 T-DNA, none of which tested positive.

In light of the zero detected incidence of FP967 T-DNA in the CDC Flax Breeding Program breeding lines, the current GM testing protocol consists of testing 1) all individual plants used in crosses, 2) all individual plants grown in growth chambers for increasing seed volumes for the field, 3) a random selection of 10% of the single plant selections for advancement in any year, and 4) all breeder seed leaving the program for seed growers and advanced breeding lines in regional cooperative trials or populations for winter nursery increase.

### Determining FP967 T-DNA sequence

There were several reasons to determine the sequence of the FP967 T-DNA. A complete sequence of the original construct was not required for registration when FP967 was developed, and providing this information would help fill a knowledge gap with respect to this deregistered variety. A complete sequence of the construct could also lead to the development of other event-specific assays as well as more specific or robust construct-specific assays; the current commercial assay contains a primer located in the dihydrofolate reductase (DHFR) region of *E. coli* integron 2, and shares similarity to the flax orthologue. In addition, we wanted to try to identify the flanking regions adjacent to the LB of the T-DNA to better understand the structure of this rearrangement (Figure 1B).

A large number of PCR clones were developed from the FP967 T-DNA. These were aligned against a draft version of the putative FP967 T-DNA generated from literature published about the AtALS mutant (Haughn et al. 1988; Sathasivan et al. 1990; McSheffrey et al. 1992) and cointegrate Ti plasmid construction (Zambryski et al. 1983; Fraley et al. 1985; Sanders et al. 1987). Seventy-nine sequence fragments or contigs derived from the PCR clone sequences aligned against the FP967 draft, comprising ~120 kb of sequence and covering 89% of the putative T-DNA sequence. An additional 13 fragments contained the FFS1 or FFS2 flanking regions from scaffold261 and the RB fragment of the T-DNA. The sequences of these clones has been placed in the Labarchives online repository at http://dx.doi.org/10.6070/H498851J. Several sequence gaps were present due to challenges associated with cloning long DNA fragments. In addition, plasmids containing the pBR322 ori site could not be cloned into pBluescript as these two plasmids have identical origins of replication. Several attempts at plasmid rescue from FP967 genomic DNA using PCR- and ligation-based approaches were attempted, but only flax genomic fragments unrelated to the insertion site were recovered.

NGS was used to further determine the FP967 T-DNA sequence. Both mate-pair (MP) and paired-end (PE) sequencing were used. The four lanes of MP reads (2.3 and 2.8 kbp fractions) and seven lanes of PE data were assembled against a putative T-DNA sequence using Bowtie2 (Boisvert et al. 2010). Another approach was to extract T-DNA specific sequences from the NGS reads. Reads not aligning to the *L. usitatissimum* reference sequence (Wang et al. 2012) were collected using Bowtie2 and *de novo* assembled using Ray (Langmead and Salzberg 2012) or SOAPdenovo. Contigs containing known FP967 T-DNA sequences were identified from this assembly and aligned against the draft sequence. Using these two approaches, approximately 32,000 reads aligned against the FP967 T-DNA as well as the cloned PCR fragment sequences (available at DOI: http://dx.doi.org/10.6070/H498851J). A putative structure for the T-DNA was developed (Figure 1C and supplementary data online) using these data. Further cloning work using primers designed on either side of gaps in the putative sequence was performed.

T-DNA components apparent in the putative sequence (Figure 1C) include the entire LB region with an adjacent ampicillin resistance gene from pBR322, a large fragment incorporating the *NOS* gene, the spectinomycin resistance/DHFR from *E. coli*, a chimeric *NPTII* gene (with *NOS* promoter and terminator), and the AtALS gene with an adjacent LIH (left inside homology) region from the T-DNA. Both of the repeated pBR322 fragments and the three *NOS* promoter and terminator regions were challenging to resolve, even with the NGS data. Attempts to identify the junctions of the *NOS* terminators and promoters and the pBR322 sections required manual analysis and alignment of putative contigs.

Using two assembly processes (one using unpaired MP reads aligning to known DNA fragments in the T-DNA and the other using paired MP reads that did not align to the flax reference genome) resulted in two opposite orientations for the T-DNA components between the LB and RB regions. The orientation of this central part of the T-DNA (from one of the *NOS* genes to the LIH) was resolved by examining the sequence of the PCR clones spanning the junctions of this segment. The order of the components along the T-DNA suggests the homologous recombination that introduced the intermediate cloning vector, pGH6 (Haughn et al. 1988), into the disarmed Ti-plasmid, pGV3850 (Zambryski et al. 1983), occurred between the pBR322 fragments, rather than at the LIH. This is expected as the LIH in pGH6 is derived from an octopine Ti plasmid, pTiA6, and does not share homology with the LB region present in pGV3850 (Fraley et al. 1985).

**Figure 3:**
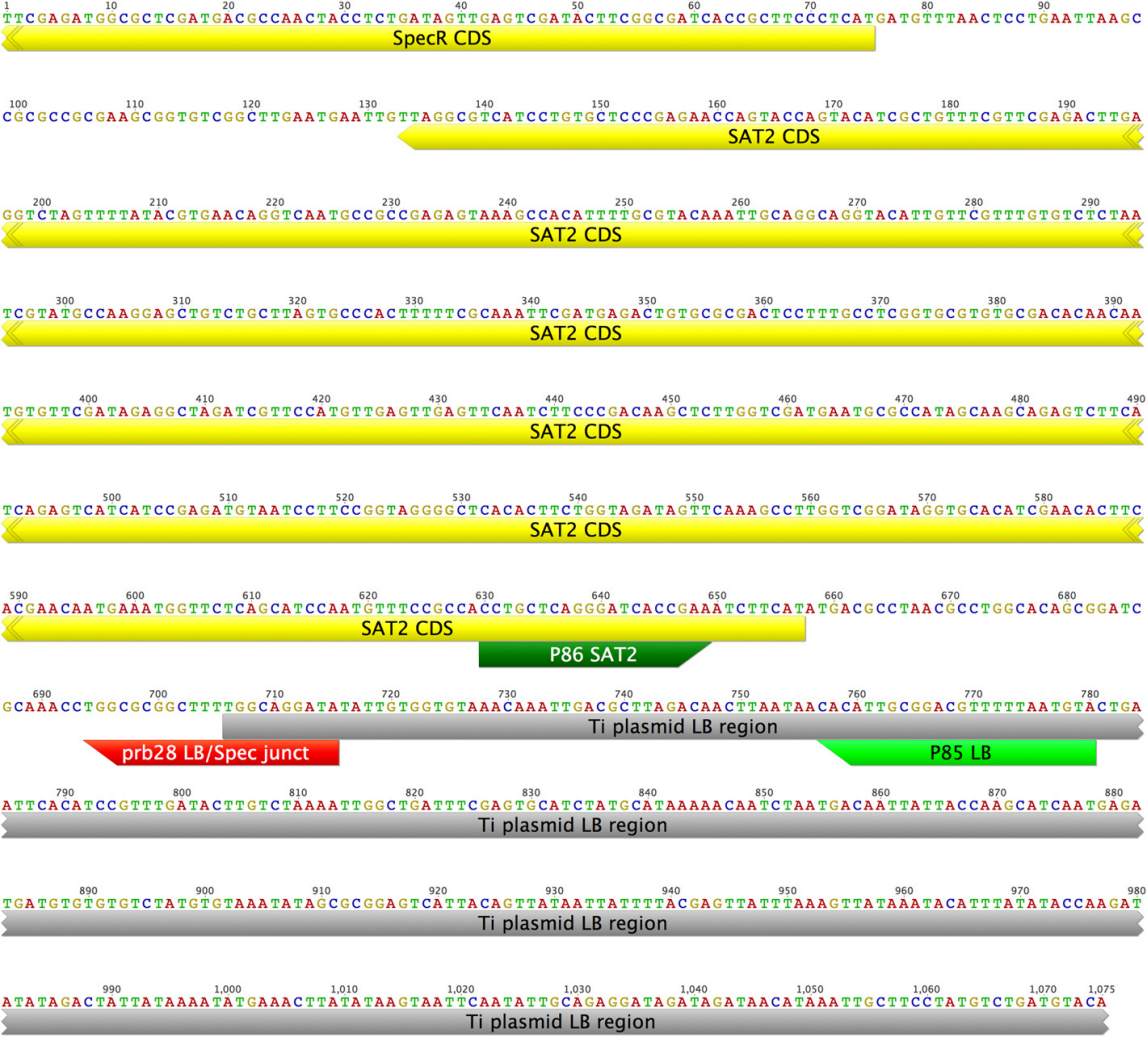
**Sequence adjacent to the LB region in FP967 and SAT2-LB event specific assay.** Our analysis indicated a region of the T-DNA between the *NOS* promoter of *NPTII* and *SAT2* was duplicated between the FFS1 region of the flax genome and the LB of the T-DNA. A qPCR assay was developed to detect this event specific fragment (P85, P86 and prb28). The sequence of these primers and probe is indicated with green and red blocks, respectively. The location of the Spectinomycin resistance gene *AADA* and *SAT2* are shown in yellow and the LB region in grey. The sequence of this fragment was confirmed by PCR sequencing of P30-P85 and P30-P20 fragments.

Evidence suggests a repeat of an internal portion of the T-DNA is located between FFS1 and the LB of the T-DNA (Figure 1C). The repeated fragment starts at the *NOS* promoter of the Nos gene and extends to just past the *streptothricin acetyltransferase 2* (*SAT2*) gene. (This gene is incorporated in the fragment of DNA used to provide streptomycin resistance (*aminoglycoside adenyltransferase A* (*AADA*)) in the intermediate cloning vectors (Fraley et al. 1983).) The evidence supporting this possibility includes six PCR clones obtained using primers located in FFS1 and near *AADA* and an assembly contig that contains 184 bp of LB adjacent to the *SAT2* gene. In addition, PCR reactions using one primer located in the duplicated region (P30 or P86) and another in the LB (P20 or P85) DNA yielded fragments of the expected size. Sequencing of these fragments revealed the expected sequence (Figure 3). The sequence of ~1 kb of this junction region is archived in the supplementary data (http://dx.doi.org/10.6070/H498851J). Together, these data show that a duplication of a portion of the T-DNA resulted in the generation of the two RB regions with flanking FFS regions and an additional copy of the chimeric *Nos* to *SAT2* fragment. This event prevented the LB from having flanking sequences in the flax genome. We estimate that the duplicated region between FFS1 and the LB (from the chimeric *NPTII* to the *SAT2/AADA* fragment) is 3,720 bp in length.

The presence of this repeat was used to develop a simple assay, SAT2-LB, that we assume is event specific as it is highly unlikely that a similar rearrangement and duplication would have occurred in other transformations. The assay developed contains a primer in the *SAT2* gene (P86), one in the LB region of the T-DNA (P85), and a probe that spans the junction between these two components (prb28; Figure 1C). This qPCR assay only detects FP967 and not Norlin gDNA, even at a low stringency (54° annealing/extension in a two-step reaction). The assay was also tested on crude gDNA extractions from seedling hypocotyls and mixtures of DNA extracted from a single FP967 seed along with nine CDC Bethune seeds. The event-specific assay was able to detect the FP967 T-DNA in both of these admixtures (data not shown).

## Conclusions

An event-specific assay was developed to detect FP967 T-DNA based on the inverted repeat structure of the two right border sequences adjacent to FFS1 and FFS2, two flax genomic DNA regions. Using the assay, we determined the existence of a single locus for the FP967 T-DNA in both F2 and BC populations. In addition, we identified FP967 T-DNA fragments in seed obtained from CDC flax breeder seed at levels of detection between 0.01 and 0.1%. Breeding lines currently advancing through the CDC Flax Breeding Program are free of the FP967 event. To determine the structure of the FP967 T-DNA, PCR cloning and NGS were utilized to identify the order of genes along the T-DNA and the sequence of most of the construct. Evidence points to a repeat of an internal T-DNA fragment, which led to the unusual inverted-repeat structure of the right border/FFS regions. The unique sequence at the T-DNA LB junction was used to design a second event-specific assay, SAT2-LB, that is specific to this site and is simpler than the one using primers situated in the flanking genomic DNA sequences.

## Abbreviations

AADA: Aminoglycoside adenyl transferease A
AtALS: *Arabidopsis* acetolactate synthase
CDC: Crop Development Center
CDS: Coding (DNA) sequence
DHFR: Dihydrofolate reductase
FFS: FP967 flanking sequence
LB: Left border
LIH: Left inside homology
MP: Mate Pair
NGS: Next generation sequencing
NTPII: Neomycin phosphotransferase II
PCR: Polymerase chain reaction
PE: Paired end
qPCR: Quantitative polymerase chain reaction
RB: Right border
SAD: Stearoyl-acyl carrier protein desaturase
SAT2: Strepothricin AcetylTransferase 2
T-DNA: Transfer DNA
